# A randomized, blinded study of photobiomodulation in a mouse model of Alzheimer’s disease showed no preventive effect

**DOI:** 10.1038/s41598-023-47039-2

**Published:** 2023-11-14

**Authors:** Mélanie Sipion, Filipa M. Ferreira, Jules Scholler, Corinne Brana, Michalina Gora, George Kouvas, Gael Barthet, Aleksander Sobolewski

**Affiliations:** Wyss Center for Bio and Neuro Engineering, Chemin des Mines 9, 1202 Geneva, Switzerland

**Keywords:** Alzheimer's disease, Neurodegeneration, Biomedical engineering

## Abstract

Photobiomodulation (PBM), the process of exposing tissue to red or near-infrared light, has become a topic of great interest as a therapy for diverse pathologies, including neurodegenerative disorders. Here, we aimed to evaluate the potential beneficial effect of PBM on Alzheimer’s disease (AD) using behavioral and histological readouts from a well-established transgenic murine AD model (5xFAD mice) in a randomized and fully blinded long-term in-vivo study following GLP (Good Laboratory Practices) guidelines. The heads of the mice were illuminated with no (sham), low or high power 810 nm light, three times a week for 5 months from the first to the sixth month of life corresponding to the prodromal phase of the pathology. The results showed that there were no significant differences between the groups in behavioral tests, including the Morris water maze, novel object recognition, and Y-maze. Similarly, histological analyses showed no differences in amyloid load, neuronal loss or microglial response. In conclusion, under the conditions of our experiment, we were unable to demonstrate any therapeutic effect of PBM for AD. This study calls for further evidence and caution when considering PBM as an effective treatment for AD.

## Introduction

Photobiomodulation (PBM), also known as low-level light therapy (LLLT), has become a topic of interest in the field of health and medicine. It is a proposed form of therapy that involves exposing tissue to light from (low-power) lasers or light-emitting diodes (LEDs). The wavelengths used for PBM are generally between 600 and 1000 nm and can vary considerably from one study to another.

This type of light exposure is claimed to ameliorate organ or cellular function in pathology, but the mechanism of action is not fully understood. It is generally accepted that it involves a photochemical reaction dependent on photoexcitation, the mechanism of electron excitation by photon absorption; in the context of PBM, the absorption of photons by biomolecules in the illuminated tissue should lead to a chemical or physical reaction involving the excited biomolecules. The exact possible candidate mechanisms are several^1,2^ and could involve processes at the molecular level such as photoisomerization (such as that undergone by retinal, the chromophore of opsins), or on a larger scale, such as enhanced fluid perfusion/microcirculation^3–6^. However, the mechanism that has received the most support involves photon absorption by cytochrome C oxidase, an enzyme of the mitochondrial respiratory chain to produce ATP^7–9^, although this hypothesis is debated^8,10–12^.

In neurology, PBM treatments for neurodegenerative and other brain diseases have gained interest recently, with reports of beneficial effects on neuropathology, proteinopathy and synaptic alterations in mouse or non-human primate models^1,2,13–20^. However, despite overwhelmingly positive outcomes reported in literature, PBM has also faced controversy in general^21–23^ and in the neurodegenerative field in particular^24^, while many studies in this field can be seen as methodologically challenged as not being fully blinded^25–27^.

Here we present a randomized and fully blinded study performed following GLP (Good Laboratory Practices) guidelines. We applied the PBM treatment in a paradigm similar to De Taboada et al.^15^ and Purushothuman et al.^19^, which can be considered the establishing studies in PBM for Alzheimer disease (AD). Specifically, we illuminated shaved heads of 5xFAD mice, a transgenic model of AD, with a collimated 1 cm diameter beam of light of 810 nm central wavelength for 2 min 3 times a week from the first to sixth month of age, i.e., during pathology development (prodromal) phase, in an attempt to block or limit it. We split the mice into three groups in which we used different power settings of the PBM illumination: zero, 6 or 600 mW/cm^2^. We performed behavioral assessment of the experimental groups before and after the 5 months of treatment. A histological characterization of the brain tissue was performed to assess the neuronal loss, the amyloid load and microglial response.

## Material and methods

### Mice

Heterozygous 5xFAD colony founders were bought from Jackson Laboratory (MMRRC_034848-JAX, B6.Cg-Tg (APPSwFlLon,PSEN1*M146L*L286V) 6799Vas/Mmjax). The colony was expanded in-house by breeding heterozygous progeny on C57BL/6J congenic background. For genotyping, the primer sequences 5′-CGG GCC TCT TCG CTA TTA C-3′ (mutant reverse), 5′-ACC CCC ATG TCA GAG TTC CT-3′ (common) 5′-TAT ACA ACC TTG GGG GAT GG-3′ (wildtype reverse) were used^28^. All experiments were performed on homozygous 5xFAD mice of both sexes from the age of 4 weeks to 32 weeks, in compliance with the Swiss Veterinary Law Guidelines and the ARRIVE guidelines, and approved by the ethics committee of the Cantonal Veterinary Office of Geneva. Throughout their life span, mice were group housed ranging from two to five animals per cage with food and water ad libitum. Transparent individual ventilated plexiglass cages were maintained on a 12 h dark/light cycle at 22 °C in a temperature-regulated room and protected from exterior pathogens.

### Study design

Animals were assigned randomly to one of three sex-balanced groups: sham treatment (n = 19), low power PBM treatment (6 mW/cm^2^, n = 20) and high power PBM treatment (600 mW/cm^2^, n = 21). Sham treatment consisted in handling and immobilization of the mouse under the PBM device similarly to experimental group animals, but with no illumination produced by the device. The mice were subjected to a battery of behavioral tests at baseline, prior to any treatments, when they were 4.5 (± 0.5) weeks old and again after the end of thetreatment (5 months later).

### Blind study

Everyone involved in the experiments and analyses of their outcomes was blinded. To ensure equal treatment of the sham mice, the person operating the PBM device only selected a mouse ID code on a custom graphical user interface, without knowing what light power (or no light) would be emitted. The group assignment was known to our quality assurance manager and remained confidential until data collection and processing had been completed. The entire analyses methodology was made ready in the form of automated scripts which were not changed after the unblinding.

### Behavioral assessment

The behavioral tests, except the Y-maze, were based on Monteiro et al.^29^.

#### Open field

On the first day the mice were habituated to an open field arena (44 × 44 cm with 30 cm high gray non-reflective walls; Ugo Basile S.l.r., Italy). Each mouse was placed in the arena facing the wall and left to explore it for 30 min.

#### Novel object recognition test (NORT)

**Figure 1:**
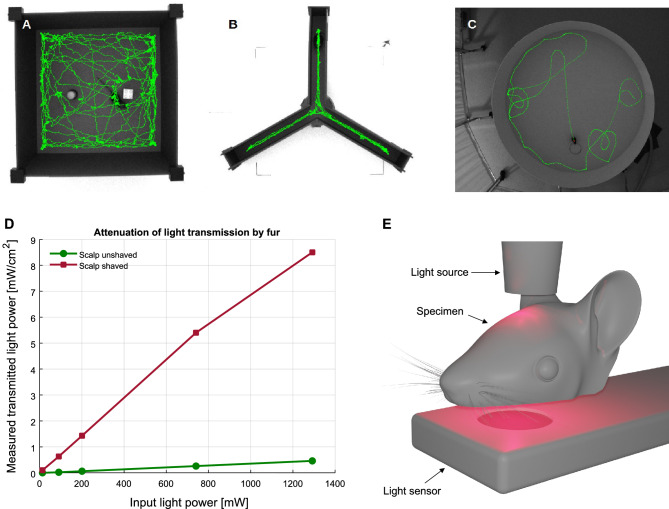
(**A**) Arena used in the NORT test, with the old (left) and novel (right) objects, overlaid with the mouse’s trajectory; animal’s increased interest in the novel object can be readily gleaned. (**B**) Y-maze used in the behavioral tests overlaid with the mouse’s trajectory. (**C**) Morris water maze used in the behavioral tests overlaid with the mouse’s trajectory on a successful trial; the outline of the submerged platform is visible through the dyed water from above, but not from water surface (mouse’s point of view). (**D**) Attenuation of light transmission by mouse fur measured ex vivo in a setup schematically depicted in (**E**).

Recognition memory was evaluated during the 2 days following the open field habituation, in the same arena. On the first day, two identical objects were symmetrically placed in the arena, at a given distance from the walls. Mice were allowed to freely explore both the objects for 10 min, after which they were returned to their home cages. Twenty-four hours later, the probe session was carried out: one of the objects was replaced by a novel one (similar size and texture but different color and shape). Each mouse was again given 10 min to explore the objects (Fig. 1A). The discrimination index, i.e., time exploring the novel object minus time exploring the old object over the total object exploration time, was calculated. The animal was considered to be exploring an object whenever it was facing the object with its nose within 5 cm from the object’s center.

#### Y-maze

Short-term spatial memory was measured 24 h after the NORT, by placing the mouse at the end of one of the arms of a radial maze with three arms (A, B and C; arm length 35 cm, width 5 cm, wall height 15 cm; Ugo Basile S.l.r., Italy), facing the center and letting it explore the maze for 10 min before being returned to its home cage (Fig. 1B). The ratio of spontaneous arm alternations (e.g. ABC, BCA, CAB, BAC) to overall number of arm entries minus two, gives an indication regarding working memory, as a healthy mouse is expected to remember the arm of the maze that it previously emerged from and will show a tendency to enter the less recently visited arm.

#### Morris water maze

To assess spatial reference memory, mice were placed in a white circular pool (120 cm diameter, 60 cm high, Ugo Basile S.l.r., Italy) filled with water at 25 ± 1 °C, made opaque with non-toxic odorless white dye (Guage Eco by Caran d'Ache SA, Switzerland). Spatial cues were placed on the walls around the pool (signs displaying a square, stripes, triangle and a cross printed in A4 format). The pool was divided into four imaginary quadrants and a transparent escape platform (10 cm diameter, 30 cm high, Ugo Basil) was placed in one of the quadrants submerged 1 cm under the water’s surface. The mice had to learn the position of a hidden platform over a period of 4 days with four trials on each day. At the beginning of each trial the mouse was placed facing the wall of the pool in a different quadrant in a pseudorandom order that varied from day to day. Each trial was completed whenever the mouse reached the platform (see Fig. 1C) or when a 120-s timeout period elapsed. On the fifth day, the platform was removed, and a single trial of 60 s was performed (probe trial) during which the accuracy of the mice’s platform seeking was quantified.

### Behavior analysis

Mice were filmed during behavioral experiments using a Zelux CS165MU/M (Thorlabs, Inc., USA) monochrome camera with a varifocal lens (model ACLV0412IR3H, Aico Electronics Limited, China). Automatic mice tracking was performed using DeepLabCut^30^ with a ResNet50 neural network. For each behavioral maze, a ResNet50 was trained on manually annotated data and then refined and validated on unseen data. All further behavioral data preprocessing and analysis was performed in Matlab (The MathWorks, Inc., USA) and Prism (Dotmatics, USA).

### Photobiomodulation setup

PBM was performed using a custom optical system consisting of an M810L4 LED (Thorlabs, Inc., USA) collimated into a homogenous ~ 1 cm diameter beam using a f20.1 mm aspheric lens (model ACL-DG6-B, Thorlabs, USA) placed approximately 15 mm from the LED surface and a f25.4 mm bi-convex lens (model LB1761-B, Thorlabs, Inc., USA) placed approximately 13 mm from the first lens. A black cone with a 1 cm opening was 3D printed to further ensure that the light was only applied to the mouse head and to help center the mouse head in the NIR light beam which is invisible to humans. Mice were immobilized in a custom-designed restraining cylinder. The peak power of light emitted by the device was set to 470 mW (600 mw/cm^2^, high power PBM condition) or 5 mW (~ 6 mw/cm^2^, low power PBM condition) based on the parameters used by Oueslati et al.^31^ and by De Taboada et al.^15^, respectively. The light was pulsed at 100 Hz with a 20% duty cycle.

### Immunostaining

Immunohistochemistry was performed on mouse brain sections. Mice were first anesthetized by an intraperitoneal injection of pentobarbital (50 mg/kg body weight) and perfused intracardially with 0.9% saline solution for 1 min and then with 4% paraformaldehyde in 0.15 M sodium phosphate buffer (PFA 4%) for 4 min. Brains were dissected, post-fixed overnight in PFA 4% followed by a cryoprotection into 30% sucrose in PBS for 24 h and then frozen within isopentane at − 55 °C cooled in a SnapFrost^®^ (Excilone, France). Coronal sections (25 µm) were cut on a Leica CM3050S Cryostat (Leica Microsystems GmbH, Germany) at − 20 °C. Sections were collected from the prefrontal cortex to the beginning of the cerebellum and stored in an antifreeze solution (0.2 M sodium phosphate buffer, glycerol 25%, ethylene glycol 30%) in 96-well plates at − 20 °C.

Prefrontal cortex and hippocampus sections from all mice were processed for multiple staining following protocol described below. Brain sections (25 µm thickness) were incubated in a multi-well plate with constant agitation. Plaques were detected by either antibodies or by Methoxy-X04 (Bio-techne, 4920/50); microglia and neurons were detected by antibodies in a multi-well plate as described in Table 1.

**Table 1:** List of staining conditions.

Microglia and plaques	Neurons	Plaques
Iba1 and methoxy-X04	NeuN	Aβ1-16 + methoxy-X04

Briefly, the sections were rinsed at room temperature (RT) in TBS (10 mM Tris pH 7.6, 0.9% NaCl) (3 × 10 min) and then incubated in a blocking solution of TBS with 0.1% Triton and 5% bovine serum albumin (BSA) for 1 h. Sections were incubated overnight at 4 °C with primary antibodies listed in Table 2.

**Table 2:** List of primary antibodies.

Antibody	Company	Reference	Species	Dilution
Aβ1-16 (6E10)	BioLegend	803003	Mouse	1:500
Aβ1-16 (DE2)	Merck	MAB5206	Mouse	1:500
Iba1	IGZ instruments	WA3 019-19741	Rabbit	1:1000
NeuN	ThermoFisher	PA578639	Rabbit	1:1000

After three washes, sections were incubated with the secondary antibody diluted 1/1000 for 2 h at RT. After washing, some sections (see Table 1) were incubated 15 min at RT with Methoxy-X04. Sections were mounted on slides in VECTASHIELD^®^ Vibrance Antifade Mounting Medium (Reactolab, H-1700-10). Brain sections were mounted on slides in VECTASHIELD^®^ Vibrance Antifade Mounting Medium with DAPI (Reactolab, H-1800-10).

### Image acquisition

Slides were loaded into a customized version of a digital slide scanner TissueScope LE120 from Huron Digital Pathology, consisting of a dual camera setup for brightfield and fluorescence imaging. Emitted fluorescence was collected by a 20×, 0.75NA WD 1mm objective and imaged on a Teledyne Photometrics–Kynetics camera. Raw data were down-sampled by a factor 2 yielding an isotropic pixel size of 0.6 μm. Settings for acquisitions and experimental parameters were kept constants for all sections stained with the specific antibody to avoid biases in fluorescence intensity.

### Histology data analysis

Stained sections were automatically analyzed using a custom pipeline written in Python in two distinct brain regions: prefrontal cortex and dorsal hippocampus. These regions were manually annotated by an expert using a custom Napari plugin. Damaged regions or regions exhibiting artifacts were excluded from further analysis. Plaques were segmented on methoxy-X04 stained images using iLastik (with all features included) that was previously trained and validated on manually annotated data. Neurons were segmented on NeuN stained images using Stardist algorithm^32^. For quantifying the microglial response to plaques, the ratio between the pixel intensity (for Iba-1 staining) close to the plaque and far from the plaque was computed. To do so, the plaque centroid and radius was extracted from the methoxy-X04 channel. The pixel intensity on Iba-1 channel was then averaged in the annulus between the plaque edge and 12 μm away from the plaque edge (i.e. close to the plaque), and in the annulus between 18 to 60 μm from the plaque edge (i.e. far from the plaque). The normalized ratio between the average Iba-1 intensity close to, and far from, the plaque was then calculated to estimate the microglial response to plaques. If microglia density was higher close to the plaque, then the ratio should be higher than 1 and if the density is similar then the ratio should be close to 1.

### Statistical analysis

Statistical analyses were performed with Matlab (The Mathworks, Inc., USA) and Prism (Dotmatics, USA). For variables with more than two levels (groups), such as the main inter-group results (sham vs. low vs. high power PBM), first the normality of data set was confirmed using the Lilliefors test. Given data being normally distributed, one-way ANOVA (analysis of variance) was performed. For variables with two levels (such as baseline vs. endpoint behavioral comparison) either paired Student’s t-test or—for non-normal data according to the Lilliefors test–Wilcoxon’s signed rank test was used. The *n* values can be found in the figure legends and correspond to the number of mice analyzed. Results are presented as mean ± SEM (standard error of the mean) unless stated otherwise. Statistical differences were considered significant at p < 0.05; denoted by one asterisk in figures; two of three asterisks denote p < 0.01 or p < 0.001, respectively.

## Results

To evaluate the potential therapeutic effect of PBM for AD, we assessed the behavior and brain histology of 5xFAD mice which, compared to other models in the literature^33^, display more severe disease progression, with brain gliosis and Aβ plaques deposition observed as early as 2 months old and behavioral cognitive impairments observed from 5 months of age. The mouse heads were illuminated by low (5 mW = 6 mW/cm^2^ peak power) or high (470 mW = 600 mW/cm^2^ peak power) power 810 nm light pulsed at 100 Hz with a 20% duty cycle thrice a week for two minutes during five months, from the first to the sixth month of life, against a control group receiving sham treatment. The low and high-power parameters are comparable to those used by Oueslati et al.^31^ and De Taboada et al.^15^, respectively, two of the landmark studies in the field of PBM for Alzheimer’s disease (AD).

We sought to verify the importance of shaving the scalp of mice in a series of ex vivo experiments on cadavers preceding the main study (Fig. 1A). We shone the light from an 810 nm light at various powers on the top of a mouse head preparation with the dorsal surface of the brain exposed to a light power meter with a 1 cm diameter sensor (model PM16-130, Thorlabs, Inc., USA) before and after shaving the scalp. The intensity of light transmitted through the brain (detected by the sensor placed under the brain after dissection of the palate) in case of unshaved heads was only 3.83 (± 1.63, depending on power) of the light transmitted when the head was shaved (Fig. 1A). This overwhelming ~ 96% attenuation of the light by the fur clearly indicates the importance of shaving the mouse scalp—an aspect not typically considered in many studies in the field^13,16–18^; possibly this effect is less pronounced in white-furred mice, but we have not tested that (our mice being black-furred). Given the above findings, the heads of the animals taking part in our study were kept shaven. The shaving was performed under a short (~ 4 min) light isoflurane anesthesia as often as needed, approximately once per week.

### Behavioral results

**Figure 2:**
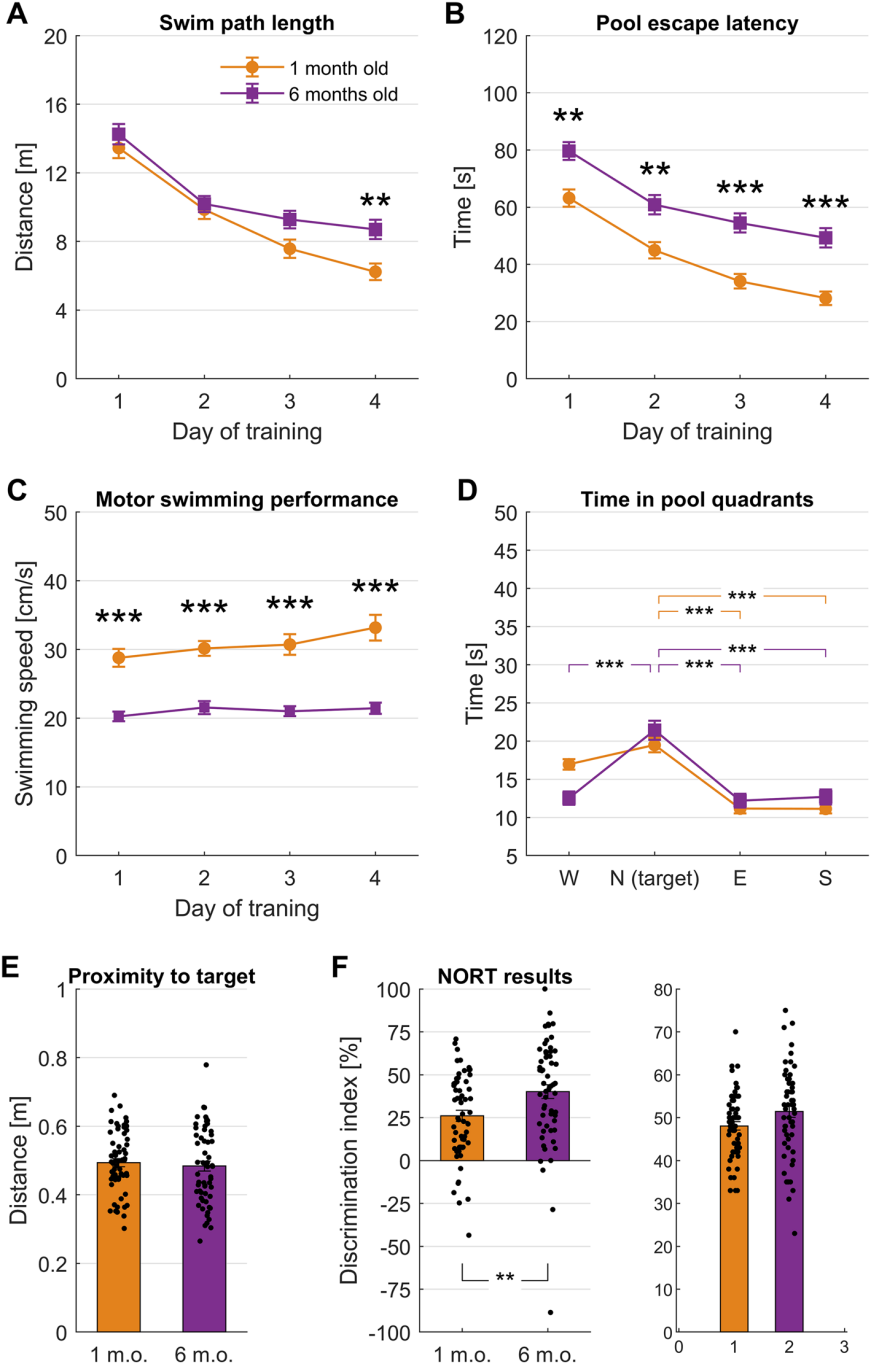
Differences in behavioral performance between baseline (1 m.o. mice, n = 60) and endpoint (same mice at 6 m.o.) measurements. (**A**) Length of swim path taken to reach the platform (or until 120 s timeout for unsuccessful trials) across the four training days of the Morris water maze. Two-way ANOVA; Day of training, F (2.5, 151.2) = 59.2, p < 0.0001; Age, F (1, 59) = 10, p = 0.002; Interaction, F (2.7, 165) = 1.7, p = 0.172. Šídák's multiple comparisons test: day 4 of training at 1-month-old compared to 6-months-old, p = 0.002. (**B**) Escape latency, i.e. time taken to reach the platform (timeout for unsuccessful trials). Two-way ANOVA; Day of training, F (2.6, 157.6) = 56.6, p < 0.0001; Age, F (1, 59) = 51.7, p < 0.0001; Interaction, F (2.8, 169.9) = 0.5, p = 0.6864. Šídák's multiple comparisons at 1-month-old compared to 6-months-old: day 1 of training, p = 0.0019; day 2, p = 0.0017; day 3, p < 0.0001; day 4, p < 0.0001. (**C**) Mean swimming speed. Two-way ANOVA; Day of training, F (2.6, 157.3) = 2.1, p = 0.1043; Age, F (1, 59) = 118.9, p < 0.0001; Interaction, F (2.6, 158.7) = 0.9, p = 0.4205. Šídák's multiple comparisons at 1-month-old compared to 6-months-old: all days of training, p < 0.0001. (**D**) Time spent by mice in each of the four pool quadrants on fifth day, i.e. probe trial. Two-way ANOVA; Quadrants, F (3, 236) = 44.1, p < 0.0001; Age, F (1, 236) = 0.002, p = 0.9670; Interaction, F (3, 236) = 5.7, p = 0.0009. Dunnett’s multiple comparisons between quadrants: all quadrants differ from the target, p < 0.0001, except North versus West at 6 months. (**E**) Mean distance of the mice to the former platform location on fifth day, i.e. probe trial. Two-tailed paired t-test, t = 0.6026, df = 59, p = 0.5491. (**F**) Discrimination index achieved by the mice in the NORT test. One sample t-test compared to zero; One-month old (t = 8.1, df = 59), p < 0.0001; 6-months old (t = 10.0, df = 59), p < 0.0001. Two-tailed paired t-test, t = 2.8, df = 59, p = 0.0073. (**G**) Spontaneous alteration ratio achieved by the mice in the y-maze test. Asterisks denote p < 0.05 (*), p < 0.01 (**) or p < 0.001 (***). Wilcoxon matched-pairs signed rank test, sum of positive, negative ranks (1316, − 700), p = 0.0344.

First, to control behavioral expression of any modelled memory deficits, we compared the mice’s performance during endpoint (six months of age) and baseline (one month old mice) tests. We used Morris’ water maze to assess long-term spatial memory, novel object recognition to assess long-term non-spatial memory, and Y-maze for short-term memory (Fig. 1B–D). As reported for the 5xFAD model^28^, we observed impeded learning in the Morris water maze. The slope of the learning curve—as measured by swim path length—flattened out at a significantly higher levels after the two first days (Fig. 2A) demonstrating inferior learning of the older 5xFAD mice in the MWM paradigm. The escape latency (time to find the platform) was significantly longer across the entire training course at six months of age compared to one month old mice (Fig. 2B). This secondary observation is, however, partially attributable also to reduced motor performance (slower swimming speed, Fig. 2C).

However, despite impaired learning, the mice’s performance on day five of the Morris water maze test (probe trial) did not differ significantly between baseline and endpoint measurements. The mice spent a comparable amount of time in the former platform quadrant (Fig. 2D) and their mean distance to the former platform location was almost identical (Fig. 2E).

We did not observe worse performance at endpoint compared to baseline in the other two tests conducted. To the contrary, on average mice exhibited significantly higher discrimination index in the novel object recognition test (Fig. 2F) and higher spontaneous alteration ratio in the y-maze test at six months of age (Fig. 2G).

**Figure 3:**
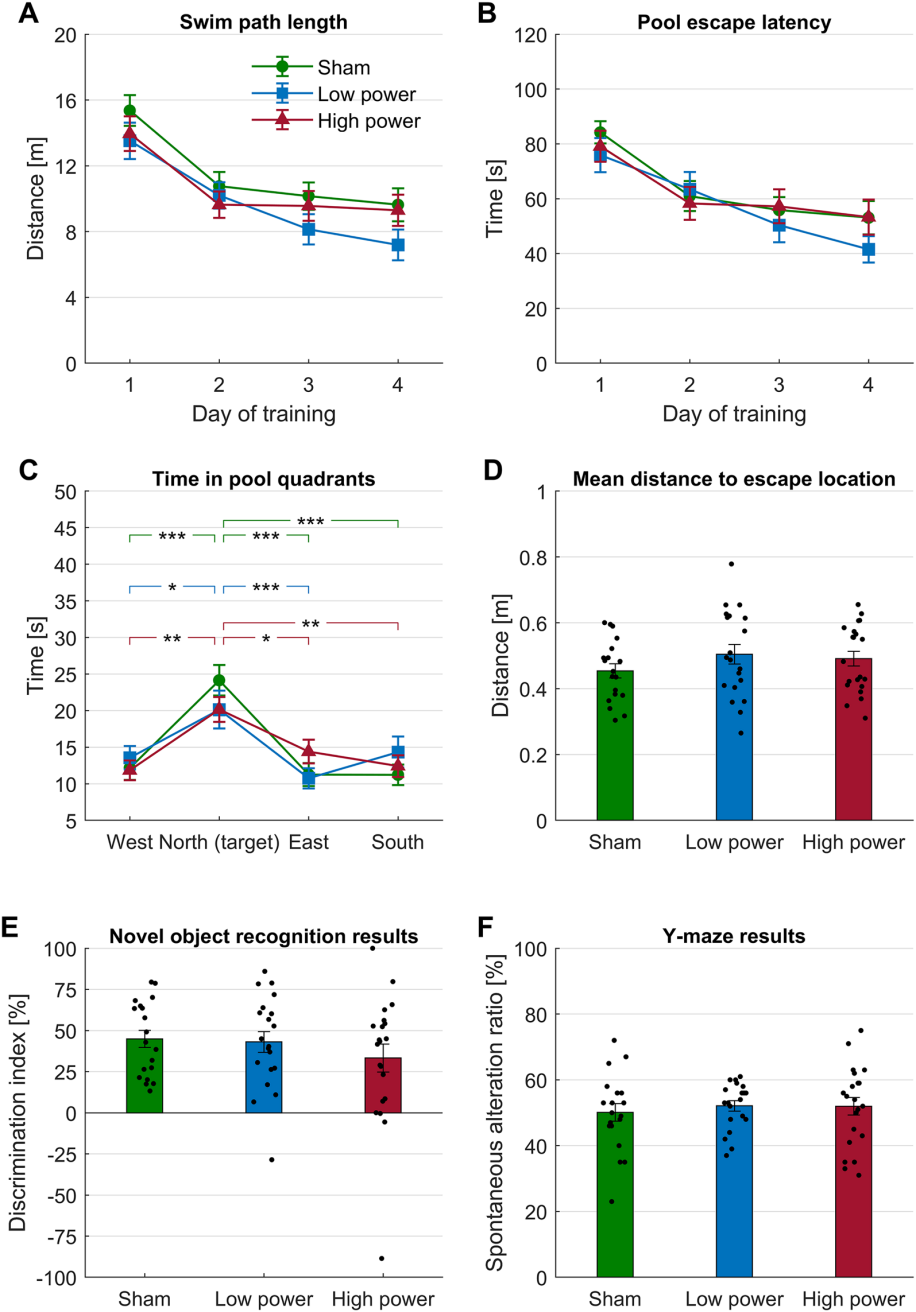
Behavioral effects of PBM. (**A**) Length of swim path taken to reach the platform (or until 120 s timeout for unsuccessful trials) for sham (n = 19), low power (n = 20) or high power (n = 21) PBM treated mice across the four training days of the Morris water maze. Two-way ANOVA; Day of training, F (2.8, 157.6) = 23.9, p < 0.0001; Treatment, F (2, 57) = 2.6, p = 0.00813; Interaction, F (6, 171) = 0.5, p = 0.8166. (**B**) Escape latency, i.e. time taken to reach the platform (timeout for unsuccessful trials). Two-way ANOVA; Day of training, F (2.9, 162.5) = 21.3, p < 0.0001; Treatment, F (2, 57) = 0.6, p = 0.5553; Interaction, F (6, 171) = 0.6, p = 0.6961. (**C**) Time spent by mice in each of the four pool quadrants on fifth day, i.e. probe trial. Two-way ANOVA; Quadrants, F (3, 228) = 20.2, p < 0.0001; Treatments, F (2, 228) = 8.10e-6, p > 0.999; Interaction, F (6, 228) = 1.4, p = 0.23. Bunnett’s multiple comparisons N (target) compared to other quadrants, p < 0.05, except North versus South at low intensity. (**D**) Mean distance of the mice to the former platform location on fifth day, i.e. probe trial. One-way ANOVA; F (2, 57) = 1.1, p = 0.3471. (**E**) Discrimination index achieved by the mice in the NORT test. One sample t-test compared to zero; Sham (t = 8.597, df = 18), p < 0.0001; Low (t = 6.8, df = 19), p < 0.0001; High (t = 3.9, df = 20), p = 0.0009. One-way ANOVA; F (2, 57) = 0.8, p = 0.4499. (**F**) Spontaneous alteration ratio achieved by the mice in the y-maze test. Lack of asterisks on the charts indicates that none of the differences between the variables shown were statistically significant. One sample t-test compared to theoretical mean = 22; Sham (t = 10.4, df = 18), p < 0.0001; Low (t = 18.8, df = 19), p < 0.0001; High (t = 11.2, df = 20), p < 0.0001. One-way ANOVA; F (2, 57) = 0.2, p = 0.8104.

We saw no differences between sham vs. low vs. high power PBM groups in any of the behavioral tests performed. As evidenced by the decreasing length of the swim path and escape latency (time to find the platform) over the four training days of the Morris water maze test, the mice—on group average—learned to some extent the location of the platform, but none of the groups was significantly better than the others on any of the days (Fig. 3A and B). On the fifth (probe trial) day, mice spent more time overall in the quadrant of the pool where the platform was located (Fig. 3C), but there was no difference between experimental groups, nor was there difference in the mean distance to the former platform location during the trial (Fig. 3D), demonstrating that mice in all groups remembered the platform position to an equal extent.

The discrimination index, the principal metric of the novel object recognition test, was positive across all groups, indicating that the mice recognized—on average—the novelty of the object introduced on the second day of the test, with no significant difference between the groups (Fig. 3E). Similarly, the spontaneous alteration ratio, the principal metric of the Y-maze test, was above chance level across all groups, indicating that the mice remembered—on average—previously visited arms of the maze, again with no significant difference between the groups (Fig. 3F).

### Histology results

**Figure 4:**
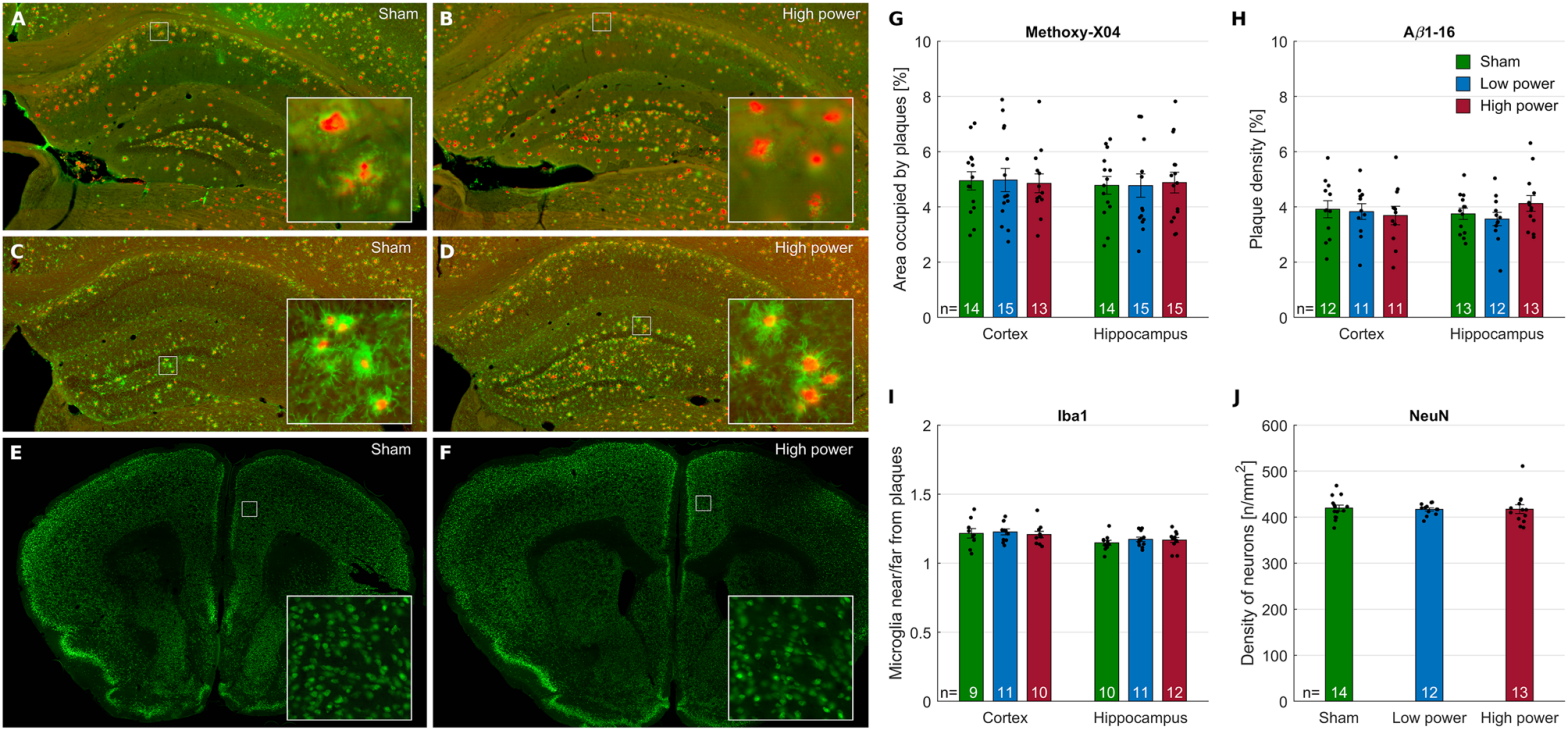
(**A**–**F**) Representative images of amyloid plaques stained with Methoxy-X04 (red channel) and Aβ1-16 (green channel) (**A**,**B**) and microglia stained for Iba-1 (green channel) around plaques stained for methoxy-X04 (red channel) (**C**,**D**) in the hippocampus, and neurons stained for NeuN in the prefrontal cortex (**E**,**F**) are depicted for sham and high-power groups. Lack of asterisks on the charts indicate that none of the differences between the plotted variables were statistically significant. (**G**–**J**) Quantified histological effects of PBM for amyloid plaque load (**G**,**H**), microglial response to plaques (**I**), neuronal count (**J**). (**G**) One-way ANOVA Cortex; F (2, 39) = 0.03, p = 0.9715. One-way ANOVA Hippocampus; F (2, 41) = 0.03, p = 0.9746. (**H**) One-way ANOVA Cortex; F (2, 31) = 0.1, p = 0.8723. One-way ANOVA Hippocampus; F (2, 44) = 0.3, p = 0.7676. (**I**) Cortex: One sample t-test compared to theoretical mean = 1; Sham (t = 6.3, df = 8), p = 0.0002; Low (t = 10.8, df = 10), p < 0.0001; High (t = 8.6, df = 9), p < 0.0001. One-way ANOVA; F (2, 27) = 0.1, p = 0.8656. Hippocampus: One sample t-test compared to theoretical mean = 1; Sham (t = 8.0, df = 9), p < 0.0001; Low (t = 10.4, df = 10), p < 0.0001; High (t = 9.2, df = 11), p < 0.0001. One-way ANOVA; F (2, 30) = 0.5, p = 0.6038. (**J**) Cortex: One-way ANOVA; F (2, 36) = 0.05, p = 0.9546.

AD is characterized by histological hallmarks, notably the deposition of Aβ peptides into amyloid plaques, the microglial response to these amyloid deposits, and neuronal loss. We assessed whether PBM treatment had an impact on these parameters. To this end, we revealed plaques in the prefrontal cortex (PFC) and hippocampus by congophilic labeling (i.e., using methoxy-X04) and by immunostaining against pan-Aβ peptides using Aβ1-16 antibody (Fig. 4A,B). There were no significant differences between sham vs. low vs. high power PBM groups in amyloid plaque load, measured by plaque area using either methoxy-X04 or Aβ1-16 labeling (Fig. 4G,H). We revealed the microglial response to plaques by co-labelling them with Iba-1 and methoxy-X04 staining (Fig. 4C,D) and by comparing the surface labelled by Iba-1 near and far from the plaques (Fig. 4I). Again, the PBM treatments had no effect regardless of the intensity used. Finally, PBM treatments did not alter the numbers of neurons in the PFC revealed by immunolabelling against NeuN (Fig. 4E,F,J).

## Discussion

Over the years the interest in using PBM as a treatment for complex neurological diseases such as Parkinson’s disease and AD has increased considerably, driven by a series of promising studies in rodents^1,2,13–15,19^. However, some skepticism remains related to PBM’s mechanism of action, as well as the true efficacy of such treatments^21–24^. Hence, with this study we aimed at shining light on the potential effectiveness of PBM to treat AD. To accomplish this, we evaluated the efficacy of PBM in a widely used and generally well accepted model of AD, the 5xFAD mice. We based our treatment schedule on the most cited study in the field^15^. We used female and male mice and initiated treatment prior to pathology onset, as it has been shown that PBM is less effective if applied after pathology’s establishment^34^. We treated mice three times a week for 120 s over a period of 5 months with 810 nm light pulsed at 100 Hz with a 20% duty cycle and peak power of 6 and 600 mW/cm^2^—comparable to the parameters used by Oueslati et al.^31^ and by De Taboada et al.^15^, respectively. In our study, even though both treatments were applied transcranially, the selected power values (low power light at 6 mw/cm^2^ and high power light at 600 mw/cm^2^) were chosen to roughly correspond to the two conditions in which light could be delivered to human brain: either transcranially, through the scalp/skull, which greatly decreases the power reaching the areas of interest (low power condition) or directly into the region of interest via an implantable device (high power condition). In the high-power condition, our ex vivo bench test results indicated that ~ 9 mW/cm^2^ of light passes through the skin/skull and reaches the dorsal surface of the mouse brain meaning that the light also reaches overlaying deep brain structures such as the hippocampus. In this study, the use of LED instead of laser follows the recent evolution in the field towards a technology that is more economical, safer and with higher potential for translation to human therapies (see^35^ for a review). At six months of age, after completion of PBM treatment, we observed no differences in behavioral performance between the sham and treated groups. These results differ from the results of the De Taboada study, in which it is shown that latency in the MWM halves in the group treated with pulsed light at 566 mW/cm2 power when compared to sham group. The use of a different mouse line in our study should not justify this difference as the two models develop plaques and behavioral impairments at approximately the same age (“Research Models Search | ALZFORUM,” n.d.). Moreover, in our study we do not see differences in amyloid load between the different groups in the cortex or hippocampus, suggesting PBM is not effective at reducing plaques in the brain of 5xFAD mice.

A caveat of our study is that we have not included wildtype mice as control. The reason for that choice was twofold: amyloid beta is known to not accumulate in the brain of healthy control mice, and the cognitive (behavioral) decline observed in 5xFAD seemed very well established in the literature. The 5xFAD mice used in our study clearly exhibited the first effect (pathological amyloid beta accumulation). We cannot, however, conclude that we fully observed the expected cognitive (behavioral) decline: older mice indeed learned worse in the MWM test, but in fact performed better in the other two tests. This outcome suggests that 6 months of age may be too early for behavioral assessment on this model or that other, more sensitive tests that better discriminate small cognitive changes are needed. Importantly, in comparison to the human progression of the disease, this would place our study as targeting the mild cognitive impairment (MCI) stage of AD, a phase when human patients show only small signs of cognitive disfunction but already present widespread pathology in the brain. MCI has, in fact, been highlighted in past years as the crucial point for treatment introduction. Indeed, the most recently approved drugs for AD were marketed to target MCI, and were approved despite minimal clinical improvement, due to a clear reduction in amyloid burden used as a pathology surrogate^36,37^. Following this logic, for PBM to become a treatment option for AD in humans, it would be necessary that it modifies disease manifestation in terms of brain pathology. However, under the conditions of our experiment—which were similar to some of those previously found to be effective—PBM did not alter the course of AD-like pathology.

## Data Availability

The datasets used and/or analysed during the current study are available from the corresponding author on reasonable request.
